# “Let's Talk about Sex”: A Qualitative Study of Rwandan Adolescents' Views on Sex and HIV

**DOI:** 10.1371/journal.pone.0102933

**Published:** 2014-08-05

**Authors:** Jennifer Ilo Van Nuil, Philippe Mutwa, Brenda Asiimwe-Kateera, Evelyne Kestelyn, Joseph Vyankandondera, Robert Pool, John Ruhirimbura, Chantal Kanakuze, Peter Reiss, Sibyl P. M. Geelen, Janneke H. van de Wijgert, Kimberly R. Boer

**Affiliations:** 1 Rinda Ubuzima, Kigali, Rwanda; 2 Department of Global Health and Amsterdam Institute for Global Health and Development, Academic Medical Center, Amsterdam, The Netherlands; 3 Department of Pediatrics, Kigali University Teaching Hospital, Kigali, Rwanda; 4 Department of Obstetrics and Gynecology, King Faisal Hospital, Kigali, Rwanda; 5 Center for Social Science and Global Health, University of Amsterdam, Amsterdam, The Netherlands; 6 Treatment and Research on HIV/AIDS Center, Kigali, Rwanda; 7 Utrecht University Children's Hospital, University Medical Center Utrecht, Utrecht, The Netherlands; 8 Biomedical Research, Epidemiology Unit, Royal Tropical Institute, Amsterdam, The Netherlands; London School of Hygiene and Tropical Medicine, United Kingdom

## Abstract

**Objective:**

This qualitative study explored the views and experiences of adolescents with perinatally acquired HIV in Kigali, Rwanda, regarding sex, love, marriage, children and hope for the future.

**Design:**

The study enrolled 42 adolescents who had received combination antiretroviral therapy for at least 12 months, and a selection of their primary caregivers. Study methods included 3 multiple day workshops consisting of role-playing and focus group discussions (FGDs) with adolescents, 8 in-depth interviews with adolescents, and one FGD with caregivers.

**Results:**

The adolescents reported experiencing similar sexual needs and dilemmas as most other adolescents, but with an added layer of complexity due to fears related to HIV transmission and/or rejection by partners. They desired more advice from their parents/caregivers on these topics. Although they struggled with aspects of sex, love, marriage and having children, most agreed that they would find love, be married and have children in the future. The two most discussed HIV-related anxieties were how and when to disclose to a (potential) sex/marriage partner and whether to have children. However, most adolescents felt that they had a right to love and be loved, and were aware of prevention-of-mother-to-child-transmission (PMTCT) options in Rwanda. Adolescents generally spoke about their future role in society in a positive manner.

**Conclusion:**

Strengthening the life skills of HIV-positive adolescents, especially around HIV disclosure and reduction of HIV transmission, as well as the support skills of parents/caregivers, may not only reduce onward HIV transmission but also improve quality of life by reducing anxiety.

## Introduction

Thanks to the widespread availability of combination antiretroviral therapy (cART) in developing countries, HIV-associated mortality in children in sub-Saharan Africa has dropped significantly [1]–[6]. Therefore, children with perinatally acquired HIV are living longer, maturing into adolescence and becoming sexually active [7], [ 8]. Studies from both resource-rich (USA and Canada) and resource-poor (Uganda) countries have shown that most perinatally-infected adolescents know their HIV status by the time they are 12 years of age and that over 80% have their first sexual experience knowing that they are HIV infected [7]–[9]. Subsequently, in addition to coping with the normal stresses of adolescence, they have to deal with several complexities related to HIV. Such complexities include a diminished sense of sexual attractiveness, anxiety about HIV transmission, fear of getting pregnant and having an HIV infected child, and the dilemma of disclosing or not disclosing to (potential) sexual partners [10]–[12]. HIV-positive adolescents are therefore challenged with balancing their sexual exploration against concerns of protecting others from HIV transmission and social acceptance [8], [ 13].

In the African setting, sex and sexuality are rarely discussed [14], [ 15]. A study [16] in Tanzania and South Africa indicated that nearly half of HIV-infected adolescents had never talked about sexuality with their parents, adult family members or teachers. Poor parent child communication may be associated with risky sexual behavior [17]. Furthermore, most national counseling programs emphasize abstinence, and the provision of condoms is often prohibited in schools despite evidence that some adolescents are sexually active [18]–[21]. A survey in Uganda found that counseling for HIV-positive adolescents focused entirely on abstinence even though 33% of the youth reported being sexually active and 86% of those who had not yet had sex were planning to do so [21].

To better understand these challenges in our own research setting in Kigali, Rwanda, we conducted a qualitative study with adolescents who had been perinatally infected with HIV and were participating in a prospective cohort study evaluating various aspects of cART.

## Methods

### Data collection

Study participants were selected from a total of 179 HIV-infected adolescents, aged 12–21 years, aware of their HIV status, who were participating in a prospective cohort study at the HIV outpatient clinic of the Institute of HIV/AIDS and Disease Prevention and Control (IHDPC, formerly TRACPlus) in Kigali. At the time of the study, approximately 600 children and adolescents were receiving care at the TRACPlus clinic, including 444 receiving cART. Of those on cART, 384 had been receiving treatment for over 12 months and 179 of the 384 were over 12 years of age. We contacted the primary caregivers of 89 adolescents who scheduled clinic visits within the two-month time period of this qualitative study. All adolescents who were interested and available, and whose primary caregivers provided consent/assent (see below) were enrolled (n = 42). The study took place from October to November 2010, which was a school break period, to reduce the number of adolescents away at boarding school. The primary caregivers were known by clinic staff, and included parents, other family members and foster care guardians.

All study procedures were conducted in private rooms in a recreational facility several kilometers from the HIV clinic to maximize confidentiality and comfort. Adolescents participated in either a two or 3 day-long mixed-gender workshop (each workshop included a different group of adolescents; total n = 42) and a selection of caregivers participated in one mixed-gender FGD (n = 10). There were two 2-day workshops and one 3-day workshop. The workshops (referred to as FGD-1, 2 and 3) were divided into sessions by discussion topic and included the following topics: Learning one's own HIV status (disclosure), disclosure to others and related-stigma, issues related to care, treatment, and adherence, health-seeking behaviors and social support, as well as issues about children, relationships, sexual relations, and future desires. Each session started with a role-play, followed by a discussion. The purpose of the role-plays was to focus the adolescents on a topic, make them feel more comfortable with the topic and each other, and to stimulate reactions. In addition, a total of 8 in-depth interviews (IDIs) with adolescents were conducted to minimize the possibility that important information was missed due to group dynamics.

The workshops were moderated by one trained male or female moderator using a topic guide. The moderators knew the adolescents prior to this research project. The moderators were assisted by a notetaker and a study staff member in charge of the tape-recording. The IDIs were conducted by a different interviewer, who had not been part of the workshops. Participants used nicknames instead of their real names during FGDs and IDIs. The interviews were digitally recorded in Kinyarwanda, transcribed verbatim, translated from Kinyarwanda into English, and uploaded into ATLAS.ti (ATLAS.ti GmbH, Berlin, Germany) for analysis.

### Ethics Statement

The Rwandan National Ethics Committee and National AIDS Control Program approved this research. All caregivers gave written informed consent for the adolescents and for themselves (if participating in a FGD themselves), and all adolescents gave written assent (the age of consent in Rwanda is 21). The participants received the equivalent of about 7 US dollar in local currency as a reimbursement for transport and time.

### Data analysis

The IDIs and FGDs were first coded in ATLAS.ti using framework analysis, a process described by Krueger and Casey [22], in which a list of categories was derived after multiple readings of the data. In order to understand issues related to sexuality and future relationships, thematic analysis was used to regroup the codes into broader themes. We recoded and regrouped the entire dataset in successive returns to the data, based not only on the original codes but also on emergent codes. The data were coded first into two umbrella areas: ‘sexuality’ and ‘future’. The umbrella areas were further investigated and four themes emerged: 1) talk about sex; 2) disclosure in relation to sex, love and marriage; 3) children; and 4) hope for the future. All of these themes were inter-related.

## Results

Thirty-two of the 89 participants could not be contacted and 9 refused to participate. The two main reasons for refusal were that adolescents did not want to attend more clinic visits or were physically outside of Kigali during the study period. No adolescent stated that they did not want to participate due to the sensitive nature of the topics. Six adolescents were given appointments but did not attend the study workshops. The remaining 42 adolescents were included in the study. All of the adolescents reported that they were HIV-infected perinatally, which was confirmed by their caregivers. About half (48%) of the adolescents reported going for HIV testing as a child because of illness or death of one of their parents, 33% because they felt sick, the remaining (19%) underwent an HIV test because of illness or death of a sibling. All were on cART for at least 12 months and the median duration of cART was 5 years (ranging from 3 to 6 years).

The median age of the adolescents was 17 years (ranging from 12 to 21 years), 45% were females, 45% were orphans, and none were married. The primary caregivers who participated in the FGD included 6 biological parents, two other family members and two foster parents. There were 6 women and 4 men included in the primary caregiver FGD.

### Let's talk about sex

During the FGDs and IDIs, the adolescents had much to say about many aspects of sex: from sexual education to feelings about and consequences of sex. Adolescents often discussed extramarital sex using words with negative connotations, such as *ubusambanyi*, which is a derogative term for premarital sexual behavior and sexual affairs outside of marriage (Table 1, Quote 1). *Ubusambanyi* was also associated with negative consequences of sex such as acquiring HIV or other sexually transmitted infections (STIs). However, when discussing sex in the context of marriage or children, attitudes were more positive.

**Table 1:** Quotes regarding talking about sex.

Quote 1
*Young people are generally threatened by epidemics but due to their mentality, they…get involved in * ***hideous behaviors*** * under the pretext that they will seek treatment if they acquire any infection… they easily get involved in * ***disgusting deeds*** *.* (Participant FGD-3)
Quote 2
Discussion between two participants from FGD-2 regarding condom use and the promotion of premarital sex behavior:
*… when a parent tells you about the condom s/he indirectly gives you the right to have premarital sex, everybody our age learns it in primary six that having premarital sex is bad and has consequences…What I wish is that parents should teach their children about abstinence but I do not support the idea of condom.*
*My mum is a devoted religious person, she says that sex is a sin and she is averse to condoms. You cannot talk about it with her…I do not know if she even knows it exists. There are parents with whom you cannot talk about condom use, they say you are already a sinner.*
Quote 3
*I think once I have sex it may increase my infection, although I think it is not good, I sometimes feel the need to have sex.* (Participant FGD-1)
Quote 4
*If you consider prostitutes, you will find out that most of them are poor people…As for the young lady who joins prostitution while she belongs to a rich family, it is the case of a child who did not have the chance to talk about reproductive health by her parents.* (Participant FGD-3)
Quote 5
*Some say ‘it is not my mistake, if I infect somebody I do not care'. I see it at work, you pay all your attention so that you do not get infected or do not infect…some other people say ‘let me infect this one, after all, it was not my mistake'.* (Participant parent FGD)

Adolescents received information about sex from a variety of sources (school, home, church, and peers) as well as from personal experience. Most adolescents felt that they were receiving sufficient sex education from clinic staff and/or at school, but there were mixed opinions about the amount and quality of the sex education provided by parents and caregivers. These opinions ranged from adequate sex education, to limited advice, to no information at all. In general, adolescents wanted advice from their caregivers on topics related to sex before marriage, and how and when to disclose their HIV status to (potential) sexual or marriage partners. However, although some adolescents said that they were able to speak to their caregivers about these issues, many said that they were not. Furthermore, discussions with caregivers about condom use were often avoided due to the widespread belief that condom education promotes premarital sex (Table 1, Quote 2). Adolescents felt that it is the responsibility of teachers and clinicians (and in their case also researchers) to provide sex education, but that it is also the responsibility of caregivers. An adolescent from FGD-3 said, “*we [adolescents] should also welcome advice provided by parents…we should understand that their advice aims to prepare us for a good future*.”

The adolescents were aware of the ways in which HIV and other STIs can be transmitted, and most knew that HIV transmission to others could be prevented by abstinence or using condoms. In general they also knew how to use condoms, though stated several reasons why they are often not used: reduced sensation, fear of losing the condom inside the partner, lack of accessibility, and the shame involved in buying condoms. It was not clear whether this was based on hearsay or actual experience. Adolescents discussed that having unsafe sex might lead to STIs, opportunistic infections and increased HIV viral load. For example, one participant said he opted to abstain from sex, even though he experienced a physical need for it, because he was afraid that sex would make his HIV infection worse (Table 1, Quote 3). This physical need for sex was something that the adolescents were beginning to experience or that they learned from their peers. One adolescent in FGD-2 explained that this desire was similar “*to crazy dogs in a village that cannot be controlled*.” The adolescents clearly struggled with this physical need for sex and the knowledge that premarital sex is considered unacceptable and could lead to HIV transmission, HIV disease progression and other STIs.

Adolescents also spoke about the sexual behaviors of others; they spoke in third-person in reference to watching pornographic films and having sex at different times and in various places. It emerged that some adolescents thought that their HIV-negative peers engaged in more sex and had riskier sexual behavior (e.g. sleeping with sex workers) than their HIV-positive peers. In addition, the adolescents acknowledged that commercial sex exists, and discussed the types of people that they thought become involved in it: the poor or those lacking sex education (Table 1, Quote 4). There was minimal talk about people intentionally infecting others with HIV, but it was mentioned briefly in three of the four FGDs (including the caregivers FGD). The comments were not about actual cases but about hypothetical cases. It was typically discussed in the context of not accepting one's HIV status and intentionally not using condoms (Table 1, Quote 5).

### HIV and disclosure in the context of sex, love, and marriage

Adolescents spoke about future partnerships mostly in terms of marriage and the sero-status of the person they envisioned marrying (i.e. HIV-positive or negative). Some adolescents said that they would prefer to marry another person with HIV, because both partners would experience similar problems related to HIV (Table 2, Quote 1). Others spoke about how they might fall in love with someone who is not infected with HIV (Table 2, Quote 2). Most adolescents felt that they had a right to love and be loved, and that love does not discriminate against HIV. “*I would like to reassure you that every one of you has the right to love or be loved. Love is something very important*” (Participant from FGD-2). Overall, the adolescents felt that love was an important part of marriage. Yet, some of them worried that even if their partner loved them, he or she may not want to marry them after learning about the HIV status. The caregivers, however, seemed confident that their children would marry (Table 2, Quote 3).

**Table 2:** Quotes regarding HIV and disclosure.

Quote 1
*She [other participant] is not the only person who is HIV positive … thinking that she will not be able to get married, but there are many people who are HIV positive with whom she may get married. And due to the fact that you would share the same problem, you would live together in harmony better than living together with an HIV negative husband.* (Participant FGD-1)
Quote 2
*I may be [HIV] infected and have a non-infected boyfriend. He may ask me to show how much I love him by having sex or kissing him and I try to protect him in different ways. It becomes very complicated when he starts telling me about his plan of marrying me and that I feel I can't sleep or have sex with him because I want to protect him… this is normal situation where people fall in love while one is infected and the other is not, and they finally get married but I always wonder what to do if you love someone but you want to protect him at the same time.* (Participant FGD-2)
Quote 3
*My child is even planning to get married and she will be sitting in a Prado car, she plans on being married and having only one child because of the problem she has.* (Participant parent FGD)
Quote 4
*But if I loved [him] to the point that we start planning to be married, I would let [him] know [about the HIV infection], that is the time you really need to tell all the truth to each other.* (Participant FGD-2)
Quote 5
*But I really beg of you, do not tell your boyfriend or girlfriend that you are infected if s/he is not planning to get married to you. Yes, you should tell the truth, but not to anybody. Nobody here is going to get married now, do not go telling you boy/girlfriend in order to gain more affection or confidence from him/her. You may ruin your love career.* (Participant FGD-2)

Some adolescents said that one should not disclose one's HIV status unless there are marriage plans because it could be detrimental to the relationship (Table 2, Quote 4). Further, one's “*love career*” may be ruined if one disclosed to casual partners (Table 2, Quote 5). Overall, adolescents agreed that disclosure to marriage partners is important, but opinions about boy/girlfriends were mixed: “*I really beg you, do not tell your boy/girl friend that you are infected if s/he is not planning to get married with you. Yes, you should tell the truth, but not to just anybody*” (Participant from FGD-2).

### Desire to have children

The main worry about having biological children was the risk of passing HIV on to them. Some adolescents spoke about adoption as an alternative (Table 3, Quote 1); two participants declared that they would avoid having children altogether because of their HIV status. In quote 2 (Table 3), one participant was worried about having HIV-infected children and another participant reassured her/him not to worry about it because prevention of mother to child transmission (PMTCT) strategies are available in Rwanda. The adolescents were well aware of the availability of PMTCT options, which gave them hope about having biological children in the future (Table 3, Quote 3). Some adolescents put religious ideas before the fear of having infected children: the Bible says that one should get married and have children, and they thought that that edict should be followed.

**Table 3:** Quotes regarding the desire to have children.

Quote 1
*But as for having children, I think it is a very difficult problem for me. I wonder if any child I deliver will not be like me, That is the reason why I would prefer to adopt a child whom I can raise together with my partner. (Participant FGD-1)*
Quote 2
*Though I no longer think of it, I sometimes wonder if I will get married and my child will have the same problem I have [HIV-infected] and also suffer in this world. With my child asking me ‘what's up mum?’ Always troubling me. Or, will I get married and keep relaxing only with my husband without having children?*
*I would like to convince [another participant] that she should get married, because it is written in the Bible ‘have children and multiply’. God did not say ‘do not get married’! Thus, she has to create and idea of her future in her mind, without considering that the child may also get sick.* (Participants FGD-2)
Quote 3
*…since this protection [PMTCT] is possible today, there is no reason to say we will not have children…there is a possibility for a mother to give birth to an uninfected child, I think that when it's time for us to have children there will be more possibilities. I think there is no reason of not having children.* (Participant FGD-2)

### Hope for the future

Generally, the adolescents spoke about their future role in society, especially regarding education and career, in a positive manner, because each person is “*the hope for future Rwanda, [we] are the future leaders of this country, there is no reason for losing hope to the extent of denying [our]selves chances of education*” (Participant from FGD-2). Despite this, they were aware that HIV is still stigmatized and that there would be challenges. For example, one adolescent discussed inability to work due to illness, another spoke of thinking only about his HIV status and not being able to concentrate on work or studies, whilst two adolescents thought that they would be denied jobs or educational opportunities due to their HIV status. In the caregivers FGD, one parent said: *“Sometimes he is discouraged with studies to the extent of saying ‘what am I studying for?’ … And he makes efforts, but he keeps thinking about himself and the problems of the illness he has, and does not do well in class.”* Some adolescents also expressed loss of hope for the future for other reasons, such as not succeeding in finding a marriage partner, or not being able to have children for fear of transmitting HIV to these children, as discussed above.

## Discussion

Being born with HIV brings additional challenges to adolescence, especially around the time of sexual debut and when facing disclosure to potential sexual and marriage partners. Overall, adolescents in this study felt that they experienced similar sexual needs and dilemmas (e.g. condom negotiation) as most other adolescents, but with an added layer of complexity due to fears related to HIV transmission or rejection. Adolescents desired more advice from their parents or caregivers on these topics. Although they struggled with aspects of sex, love, marriage and having children, most agreed that they would find love, be married and have children in the future.

Studies conducted in Zambia [23] and Uganda [24] also found that HIV-positive adolescents expect a future that includes the same educational and career opportunities as their non-HIV infected peers, as well as marriage and having children. However, a study in Zimbabwe reported that feelings of despair, hopelessness and sense of imminent death are common among young people infected with HIV [25]. The adolescents in our study, as well as adolescents in Kenya, Namibia, and other Sub Saharan African countries, expressed the need for more life skills education and training to cope with the complexities related to sex and disclosure to sexual partners, especially from parents or caregivers [24], [26]–[28]. However, in Uganda, parents and caregivers expressed the communication difficulties related to sexuality, understanding adolescents' behavior, imparting life skills, and supporting adolescents to disclose their HIV status or live responsibly in a situation of non-disclosure [29].

Earlier studies have found that disclosing HIV status to a sexual partner often results in negative outcomes such as rejection by romantic partners and/or the community and stigma [30], [ 31]. Previous rejection, fear of rejection, and thinking about partner perceptions have often been reported as barriers for disclosing HIV status to the sexual partner [31], [ 32]. WHO recognizes disclosure as a complicated issue and recommends that “adolescents be counseled about the potential benefits and risks of disclosure of their HIV status and empowered and supported to determine if, when, how and to whom to disclose” [33]. However, these guidelines do not provide concrete recommendations on when and how to disclose to sexual partners.

Our study had two main limitations. First, it was difficult to differentiate between actual experiences and hearsay in the group discussions. For example, it was often not clear whether the adolescents themselves were sexually active and whether they had disclosed their HIV status to sexual partners or not. We assume that some but not all of the adolescents had been sexually active themselves, and even if they had been, that their real life experiences were limited. Second, the participants were receiving cART treatment in the capital city Kigali, and were research-experienced; they might therefore not be representative of all HIV-positive adolescents in Rwanda.

Our findings have important implications for HIV prevention as well as HIV treatment and care programming in Rwanda. Strengthening the life skills of HIV-positive adolescents, as well as the support skills of caregivers, may not only reduce onward HIV transmissions but also improve quality of life by reducing anxiety. Future studies could explore the benefits of HIV disclosure skills training, bearing in mind that adolescents would like to have control over how and when to disclose their status to others [23]. In addition, peer networks could be established to encourage HIV-positive adolescents to live positively, and help each other with the dilemmas that they face. In Uganda, HIV-positive adolescents who participated in such a peer network reported reduced sexual activity, increased disclosure of HIV status, improved adherence to cART, and less reluctance to discuss reproductive health issues with health care providers [34]. In the FGDs in our study, adolescents encouraged each other to remain hopeful, and discussed options regarding disclosure, future marriage and children. An organized peer network could also help adolescents facing depression, hopelessness and non-acceptance of their HIV status. All of these have been shown to be potential barriers to cART adherence and may jeopardize HIV treatment responses [35], [36], [37]. Finally, it would be useful to train caregivers and other family members of children living with HIV on how best to help them cope with HIV as they mature into adolescence.

## References

[1] Tukei VJ, Murungi M, Asiimwe AR, Migisha D, Maganda A, et al (2013) Virologic, immunologic and clinical response of infants to antiretroviral therapy in Kampala, Uganda. BMC Pediatr 13: 42–2431–13–42. 23536976 10.1186/1471-2431-13-42 PMC3616823

[2] Evans D, Menezes C, Mahomed K, Macdonald P, Untiedt S, et al (2013) Treatment outcomes of HIV-infected adolescents attending public-sector HIV clinics across Gauteng and Mpumalanga, South Africa. AIDS Res Hum Retroviruses 29(6): 892–900. 23373540 10.1089/aid.2012.0215 PMC3653371

[3] Edmonds A, Yotebieng M, Lusiama J, Matumona Y, Kitetele F, et al (2011) The effect of highly active antiretroviral therapy on the survival of HIV-infected children in a resource-deprived setting: a cohort study. PLoS Med 8(6): e1001044 10.1371/journal.pmed.1001044 21695087 PMC3114869

[4] Reddi A, Leeper SC, Grobler AC, Geddes R, France KH, et al (2007) Preliminary outcomes of a paediatric highly active antiretroviral therapy cohort from KwaZulu-Natal, South Africa. BMC Pediatr 7: 13. 17367540 10.1186/1471-2431-7-13 PMC1847430

[5] Janssen N, Ndirangu J, Newell ML, Bland RM (2010) Successful paediatric HIV treatment in rural primary care in Africa. Arch Dis Child 95(6): 414–421. 19880392 10.1136/adc.2009.169367 PMC3181433

[6] Kabue MM, Buck WC, Wanless SR, Cox CM, McCollum ED, et al (2012) Mortality and clinical outcomes in HIV-infected children on antiretroviral therapy in Malawi, Lesotho, and Swaziland. Pediatrics 130(3): e591–9. 22891234 10.1542/peds.2011-1187 PMC3962849

[7] Birungi H, Mugisha JF, Obare F, Nyombi JK (2009) Sexual behavior and desires among adolescents perinatally infected with human immunodeficiency virus in Uganda: implications for programming. J Adolesc Health 44(2): 184–187. 19167668 10.1016/j.jadohealth.2008.06.004

[8] Ezeanolue EE, Wodi AP, Patel R, Dieudonne A, Oleske JM (2006) Sexual behaviors and procreational intentions of adolescents and young adults with perinatally acquired human immunodeficiency virus infection: experience of an urban tertiary center. J Adolesc Health 38(6): 719–725. 16730601 10.1016/j.jadohealth.2005.06.015

[9] Fernet M, Wong K, Richard ME, Otis J, Levy JJ, et al (2011) Romantic relationships and sexual activities of the first generation of youth living with HIV since birth. AIDS Care 23(4): 393–400. 21271400 10.1080/09540121.2010.516332

[10] Bakeera-Kitaka S, Nabukeera-Barungi N, Nostlinger C, Addy K, Colebunders R (2008) Sexual risk reduction needs of adolescents living with HIV in a clinical care setting. AIDS Care 20(4): 426–433. 18449819 10.1080/09540120701867099

[11] Domek GJ (2006) Social consequences of antiretroviral therapy: preparing for the unexpected futures of HIV-positive children. The Lancet 367(9519): 1367–1369. 10.1016/S0140-6736(06)68584-X 16631916

[12] Battles HB, Wiener LS (2002) From adolescence through young adulthood: psychosocial adjustment associated with long-term survival of HIV. J Adolesc Heal 30(3): 161–168. 10.1016/s1054-139x(01)00341-x 11869922

[13] Mergui A, Giami A (2011) The sexuality of HIV-infected-adolescents: literature review and thinking on the unthinkables of sexuality. Arch Pediatr 18(7): 797–805. 21652188 10.1016/j.arcped.2011.04.015

[14] Izugbara C (2008) Home-Based Sexuality Education: Nigerian Parents Discussing Sex with their Children. Youth Soc 39: 575–600.

[15] Socar P (2008) “Teaching Taboo Topics without Talking About Them: An Epistemic Study of a New Approach to HIV/AIDS Prevention in Education in India.” Doctoral Thesis, Stanford University.

[16] Namisi FS, Flisher AJ, Overland S, Bastien S, Onya H, et al (2009) Sociodemographic variations in communication on sexuality and HIV/AIDS with parents, family members and teachers among in-school adolescents: a multi-site study in Tanzania and South Africa. Scand J Public Health 37 Suppl 265–74. 19493983 10.1177/1403494808086986

[17] Atienzo EE, Walker DM, Campero L, Lamadrid-Figueroa H, Gutierrez JP (2009) Parent-adolescent communication about sex in Morelos, Mexico: does it impact sexual behaviour? Eur J Contracept Reprod Heal Care 14(2): 111–119. 10.1080/13625180802691848 19340706

[18] Binagwaho A, Fuller A, Kerry V, Dougherty S, Agbonyitor M, et al (2012) Adolescents and the right to health: eliminating age-related barriers to HIV/AIDS services in Rwanda. AIDS Care 24(7): 936–942. 22292484 10.1080/09540121.2011.648159

[19] Ketting E, Winkelmann C (2013) New approaches to sexuality education and underlying paradigms. Bundesgesundheitsblatt Gesundheitsforschung Gesundheitsschutz 56(2): 250–255. 23361210 10.1007/s00103-012-1599-8

[20] Baryamutuma R, Baingana F (2011) Sexual, reproductive health needs and rights of young people with perinatally acquired HIV in Uganda. Afr Health Sci 11(2): 211–218. 21857852 PMC3158520

[21] Birungi H, Obare F, Mugisha JF, Evelia H, Nyombi J (2009) Preventive service needs of young people perinatally infected with HIV in Uganda. AIDS Care 21(6): 725–731. 19806488 10.1080/09540120802511901

[22] Krueger RA, Casey MA (2008) Focus Groups: A Practical Guide for Applied Research. 4th ed. Thousand Oaks, CA: Sage Publications

[23] Mburu G, Hodgson I, Teltschik A, Ram M, Haamujompa C, et al (2013) Rights-based services for adolescents living with HIV: adolescent self-efficacy and implications for health systems in Zambia. Reprod Health Matters 21(41): 176–185. 23684200 10.1016/S0968-8080(13)41701-9

[24] Obare F, Birungi H (2010) The limited effect of knowing they are HIV-positive on the sexual and reproductive experiences and intentions of infected adolescents in Uganda. Popul Stud 64(1): 97–104. 10.1080/00324720903427575 20087816

[25] Mavhu W, Berwick J, Chirawu P, Makamba M, Copas A, et al (2013) Enhancing Psychosocial Support for HIV Positive Adolescents in Harare, Zimbabwe. PLOS ONE 8(7): e70254 10.1371/journal.pone.0070254 23894625 PMC3720910

[26] Poulsen MN, Miller KS, Lin C, Fasula A, Vandenhoudt H, et al (2010) Factors associated with parent-child communication about HIV/AIDS in the United States and Kenya: a cross-cultural comparison. AIDS Behav 14(5): 1083–1094. 19763811 10.1007/s10461-009-9612-4

[27] Nambambi NM, Mufune P (2011) What is talked about when parents discuss sex with children: family based sex education in Windhoek, Namibia. Afr J Reprod Health 15(4): 120–129. 22571114

[28] Bastien S, Kajula LJ, Muhwezi WW (2011) A review of studies of parent-child communication about sexuality and HIV/AIDS in sub-Saharan Africa. Reprod Health 8: 25–4755–8–25. 21943095 10.1186/1742-4755-8-25 PMC3192730

[29] Kisaakye VNK, Nyombi J, Nakalawa L (2009) Designing interventions for parents/guardians of HIV perinatally infected adolescents in Uganda [abstract]. International AIDS Society Conference.

[30] Fair C, Albright J (2012) “Don't tell him you have HIV unless he's ‘the one’”: romantic relationships among adolescents and young adults with perinatal HIV infection. AIDS Patient Care STDs 26(12): 746–754. 23199192 10.1089/apc.2012.0290

[31] Greenhalgh C, Evangeli M, Frize G, Foster C, Fidler S (2013) Intimate relationships in young adults with perinatally acquired HIV: Partner considerations. AIDS Care 25(4): 447–450. 22909272 10.1080/09540121.2012.712671

[32] Wiener LS, Battles HB (2006) Untangling the web: a close look at diagnosis disclosure among HIV-infected adolescents. J Adolesc Health 38(3): 307–309. 16488835 10.1016/j.jadohealth.2005.03.024 PMC2408712

[33] WHO (2013) Consolidated ARV guidelines. Available: http://www.who.int/hiv/pub/guidelines/arv2013/intro/rag/en/index.html. Accessed 2013 Oct 2.

[34] Mukasa Mbooga B, Kawuma E, Batamwita R, Kabunga G, Kwesiga M, et al.. (2012) Responding to the sexual and reproductive health needs of adolescents living with HIV and AIDS: the Mildmay Uganda experience [abstract]. International AIDS Society Conference.

[35] Byakika-Tusiime J, Crane J, Oyugi J, Ragland K, Kawuma A, et al (2009) Longitudinal antiretroviral adherence in HIV+ Ugandan parents and their children initiating HAART in the MTCT-Plus Family Treatment Model: Role of depression in declining adherence over time. AIDS Behav 13: S82–S91. 10.1007/s10461-009-9546-x 19301113

[36] Nugent NR, Brown LK, Belzer M, Harper GW, Nachman S, et al (2010) Adolescent Trials Network for HIV/AIDS Interventions. Youth Living With HIV and Problem Substance Use: Elevated Distress Is Associated With Nonadherence and Sexual Risk. J Int Assoc Physicians AIDS Care 9(2): 113–115. 10.1177/1545109709357472 PMC3052784 20133498

[37] Mutwa PR, Van Nuil JI, Asiimwe-Kateera B, Kestelyn E, Vyankandondera J, et al (2013) Living situation affects adherence to combination antiretroviral therapy in HIV-infected adolescents in Rwanda: a qualitative study. PLOS ONE 8(4): e60073 10.1371/journal.pone.0060073 23573232 PMC3616046

